# Targeting cardiomyocyte cell cycle regulation in heart failure

**DOI:** 10.1007/s00395-024-01049-x

**Published:** 2024-04-29

**Authors:** Chaonan Zhu, Ting Yuan, Jaya Krishnan

**Affiliations:** 1 Department of Medicine III, Cardiology/Angiology/Nephrology, Goethe University Hospital, 60590 Frankfurt am Main, Germany; 2 Institute for Cardiovascular Regeneration, Goethe University, 60590 Frankfurt am Main, Germany; 3 German Center for Cardiovascular Research, Partner Site Rhein-Main, 60590 Frankfurt am Main, Germany; 4 Cardio-Pulmonary Institute, Goethe University Hospital, 60590 Frankfurt am Main, Germany

**Keywords:** Cell cycle, Cardiomyocyte proliferation, Heart failure

## Abstract

Heart failure continues to be a significant global health concern, causing substantial morbidity and mortality. The limited ability of the adult heart to regenerate has posed challenges in finding effective treatments for cardiac pathologies. While various medications and surgical interventions have been used to improve cardiac function, they are not able to address the extensive loss of functioning cardiomyocytes that occurs during cardiac injury. As a result, there is growing interest in understanding how the cell cycle is regulated and exploring the potential for stimulating cardiomyocyte proliferation as a means of promoting heart regeneration. This review aims to provide an overview of current knowledge on cell cycle regulation and mechanisms underlying cardiomyocyte proliferation in cases of heart failure, while also highlighting established and novel therapeutic strategies targeting this area for treatment purposes.

## Introduction

Heart failure is becoming a growing concern globally in industrialized societies. The prevalence of heart failure has resulted in significant mortality rates and poor prognoses for individuals affected by these conditions. Loss of cardiomyocytes, or CMs, is observed in various human heart failure including hypertensive cardiomyopathy, myocarditis, myocardial infarction (MI), and aortic stenosis. This loss ultimately leads to decreased cardiac function and potential progression to heart failure [58, 72, 93, 158]. Unlike certain amphibians and fish that can regenerate their hearts following injury [54, 111, 140], the regenerative capacity of the adult mammalian heart is limited [17, 154]. Less than 1% of adult mammalian cardiomyocytes have the ability to proliferate within one year, with this rate declining as individuals age [103]. Regrettably, lost cardiomyocytes are replaced by non-functional scar tissue that cannot be effectively restored after injury occurs [66, 141]. One of the primary reasons for the limited regenerative capacity in adult mammalian cardiomyocytes is their gradual exit from the cell cycle during maturation [68, 94]. As a result, they lose their ability to proliferate and have very restricted potential to re-enter the cell cycle.

Promoting cardiomyocytes to re-enter the cell cycle and complete cytokinesis after cardiac injury is a crucial approach to addressing heart failure. In the past four decades, numerous studies have investigated various methods of inducing cardiomyocyte cell cycle activity to enhance proliferation, including regulating cell cycle regulators, signaling pathways, non-coding RNAs (ncRNAs), and metabolism [16, 29, 45, 124]. So far, we have reached a stage of consolidation with new tools at hand, such as gene therapy and delivery methods. This review will focus on the current understanding of how cardiomyocyte cell cycle activity is regulated as a potential mechanism for cardiac repair. Based on current literature, we will also discuss targeting the cell cycle as a strategy for cardiac regeneration therapy in treating heart failure.

## The mammalian cell cycle

The cell cycle is the process in which a cell duplicates and divides into two daughter cells. In mammals, the cell cycle consists of G1 phase, synthesis (S) phase, G2 phase, and M phase (mitosis and cytokinesis) [65, 96]. During G1 and G2 phases, the cell grows and prepares for division [48, 143]. The S phase is when DNA synthesis occurs [22]. After interphase, the cell enters a checkpoint before progressing to mitosis [163]. Mitosis encompasses several phases including prophase, prometaphase, metaphase, anaphase telophase, culminating in cytokinesis [185]. If the cell exits the cell cycle in response to molecular cues during any part of the cell cycle, it enters the arrested quiescent G0 state.

**Fig. 1:**
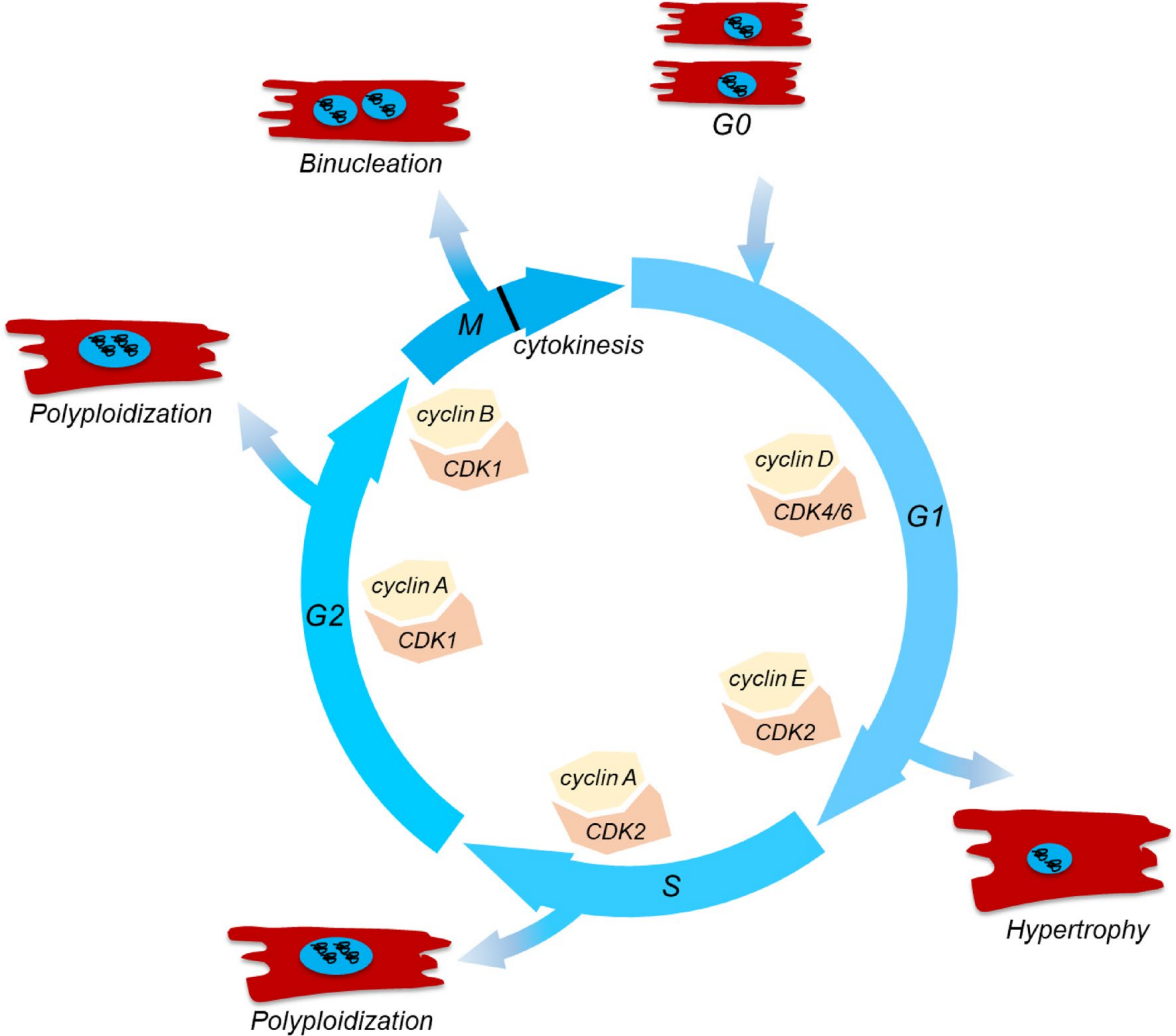
Schematic diagram of physiological and pathological processes during cardiomyocyte cell cycle progression

Cellular processes involved in the cell cycle are tightly regulated. In G1 phase, cyclin-dependent kinase 4 (CDK4) and CDK6 complexes phosphorylate retinoblastoma (RB) proteins, leading to the inhibition of E2F repressor RB and increased activity of the E2F family (Fig. 1). This enhances expression of gene driving DNA synthesis and promotes cell cycle progression [145, 162]. As the cell cycle advances towards the G1 restriction point, formation of the cyclin E-CDK2 complex occurs. This complex facilitates phosphorylation of RB proteins, stimulating G1/S transition and initiating S phase [188]. Following this transition, progression through S phase is controlled by cyclin A-CDK2 complex which regulates chromosome duplication and early mitotic events [153]. While during the G2/M transition, cyclin A forms complex with CDK1 which facilitates entry into the M phase along with cyclin B-CDK1 complex [153]. In the context of myocardial proliferation, it is suggested that targeting the regulation of the cell cycle could be a potential therapeutic approach for cardiac repair. However, due to the intricate and diverse nature of myocardial cell cycle regulation, there is still a need for more comprehensive strategies to control these processes effectively.

In mature mammalian cells, negative regulators known as cyclin-dependent kinase inhibitors (CKIs) have an important role in regulating cell cycle activity by binding to cyclin-CDK complexes and inhibiting their functions [20]. The CKI complex consists of two distinct protein groups: INK4 family members (p14, p15, p16, p18 and p19) inhibit cyclin D function on CDK4 and CDK6 leading to cell cycle arrest in G1 phase [27, 87]; while CIP/KIP family members (p21, p27 and p57) act as broad blockers preventing progression through different phases by inhibiting various cyclin-CDK complexes [40, 170]. Thus, both activating and inhibiting factors collaborate to ensure precise regulation of the cell cycle.

## Manipulation of the cell cycle to induce cardiomyocyte proliferation

In adult mammals, cardiomyocytes are specialized cells with the majority having lost their ability to divide. After birth, most of these cardiomyocytes undergo a process where they cease cell division and become polyploid and multinucleated [7, 86, 100, 161]. Polyploid and multinucleated cardiomyocytes lose the ability to proliferate and regenerate [88]. The exit from the cell cycle can occur at various checkpoints either prior to or after mitosis [8, 23, 100]. Shortly after birth, the majority of postnatal cardiomyocytes pause at the G1/S transition phase in order to prepare for their designated functions. During this transition phase, genes related to promoting cell division such as cyclins are downregulated while levels of cell cycle inhibitors like p21 and p57 increase [4, 83, 89, 137]. Although some cardiomyocytes manage to pass through the G1/S checkpoint and proceed into mitosis, most are unable to complete chromosome separation or cytokinesis resulting in polyploidy and cell cycle exit [17, 88, 94]. While the majority of adult mammalian cardiomyocytes are unable to re-enter the cell cycle, a small subset has demonstrated proliferative potential within the adult heart [6, 17], particularly in response to injury or disease [126, 154].

In recent years, numerous studies have investigated different strategies aimed at modulating the cardiomyocyte cell cycle for enhanced proliferation. A deeper understanding of the molecular mechanisms governing this process may hold promise for developing therapies that can replenish cardiomyocyte populations and restore cardiac function following cardiac injury. In this review, we will focus on the effects on cardiomyocyte cell cycle from the following perspectives: cell cycle regulators, signaling pathways, ncRNAs, metabolic pathways, epigenetic and extrinsic factors.

In the adult mammalian heart, cardiomyocytes have the ability to re-enter the cell cycle. However, this does not result in functional regeneration after injury due to the limited number of cardiomyocytes that successfully complete the cell cycle and undergo proliferation. Therefore, most research efforts focus on identifying paracrine cues that can stimulate cardiac muscle cell growth. This is particularly challenging because the majority of cardiomyocytes in adult mammals are polyploid or multi-nucleated, unlike naturally regenerative non-mammalian vertebrates. Cell cycle regulation in mammalian cardiomyocytes involves the repression of cell cycle activators, such as cyclins, CDKs, and CDK-activating kinases (CAKs), along with CKIs [23]. This leads to cardiomyocyte cell cycle arrest during cardiac development [78]. The activity of cyclin-CDK complexes is tightly controlled and plays a crucial role in facilitating cell cycle progression through phosphorylation of downstream proteins [78].

Numerous studies have been conducted to investigate the potential reactivation of cell cycle activators in cardiomyocytes. For instance, enhancing the expression of cyclin D1 has been shown to stimulate DNA synthesis in adult mouse cardiomyocytes, resulting in more than 40% of these cells re-entering the cell cycle [161, 171]. However, it should be noted that many of these stimulated cardiomyocytes experienced M-phase arrest or endoreplication. On the other hand, constitutive cardiac expression of cyclin A2 (CCNA2) in adult mice or rats leads to increased cardiomyocyte mitosis and improved cardiac function after heart injury [32, 37, 189]. Additionally, delivering the CCNA2 gene via adenovirus into peri-infarct myocardium in pig hearts promotes cytokinesis and an approximately 18% increase in ejection fraction [157]. Similarly, researchers have explored the regulation of other cyclins or CDKs, such as exogenously increasing the expression of CDK2, cyclin D2, or cyclin B1 with constitutively activated cell division cycle 2 kinase (CDC2), to enhance adult cardiomyocyte proliferation [21, 110, 133]. Additionally, specific overexpression of cyclin D2 has been found to promote cardiomyocyte proliferation, improve cardiac function, and decrease infarct size after MI in both mice and pigs in vivo [165]. Recognizing that a single cyclin or CDK may have limited impact, Mohamed et al. recently conducted combinatorial screening and reported that ectopic overexpression of CDK1-CDK4-CCNB-CCND (4F) can increase cardiomyocyte proliferation by approximately 15% to 20% in cultured mouse and human cardiomyocytes [1, 124]. Furthermore, overexpressing 4F has shown promising results by significantly increasing cardiomyocyte proliferation and enhancing cardiac function following MI in mouse models as well as rat models of subacute heart failure [1, 124].

In contrast, several studies have aimed to promote cardiomyocyte re-entry into the cell cycle by inhibiting CKIs. One study found that p16 knockdown extends the regeneration period in neonatal mice through CDK4/6 and reactive oxygen species (ROS)-related autophagy [167]. Another study demonstrated that specifically inactivating p16 in cardiomyocytes improves heart function and reduces scar size after a myocardial infarction [148]. Additionally, triple knockdown of CKIs (p21, p27 and p57) promotes cell cycle progression into the S phase and cytokinesis in adult rat cardiomyocytes, leading to an increase in their number [40]. Recently, researchers reported that silencing cyclin L1 (CCNL1) via AAV9-cTnT-CCNL1 shRNA enhances the percentage of Ki67-positive and phospho-histone H3 (pHH3)-positive cardiomyocytes in MI mice, thereby promoting cardiac repair within the infarct zone and improving cardiac function [56]. Thus, CCNL1 appears to inhibit cardiomyocyte proliferation and indicates that cyclins can have diverse, and sometimes conflicting, functions in cardiomyocyte cell cycle regulation. A summary of these findings is depicted in Table 1.

**Table 1:** Cell cycle regulators in cardiomyocyte regeneration and heart failure

Cell cycle regulators	Species	Application	Effects	References
Cyclin D1	Mouse	OE	Adult CM cell cycle reentry ↑	[162, 171]
Cyclin A2	Mouse Rat Pig	OE	Adult CM mitosis ↑ Post-MI cardiac function ↑	[32, 37, 157, 188]
Cyclin B1-CDC2	Rat	OE	Adult CM cell cycle reentry ↑	[21]
CDK2	Mouse	OE	Smaller mononuclear CMs ↑	[110]
Cyclin D2	Mouse Pig	OE	Adult CM cell cycle reentry ↑ Post-MI cardiac function ↑ Post-MI infarct size↓	[133, 165]
CDK1-CDK4-CCNB-CCND	Mouse Rat hiPSC-CM	OE	Adult CM cell cycle reentry ↑ Post-injury cardiac function ↑	[1, 124]
p16	Mouse	KD or KO	Neonatal CM regeneration period ↑ Post-MI cardiac function ↑ Post-MI infarct size ↓	[148, 167]
p21-p27-p57	Rat	KD	Adult CM cell cycle reentry ↑	[40]
Cyclin L1	Mouse	KD	Adult CM cell cycle reentry ↑ Post-MI cardiac function ↑ Post-MI infarct size ↓ Post-MI CM size ↓	[56]

*OE* overexpression; *MI* myocardial infarction; *KD* knock down; *KO* knock out; *CM* cardiomyocyte; *CDC2* cell division cycle 2 kinase; *CDK* cyclin-dependent kinase; *CCNB* cyclin B1; *CCND* cyclin D1

These studies collectively indicate that promoting cardiomyocyte proliferation can be achieved by reactivating cell cycle activators or inhibiting CKIs. However, it should be noted that certain findings demonstrate that increased DNA synthesis in cardiomyocytes may lead to multinucleation or polyploidy without complete cytokinesis. Hence, additional research is required to not only understand how to facilitate cardiomyocyte re-entry into the cell cycle but also ensure successful completion of cytokinesis.

### Manipulation of signaling pathways to induce cardiomyocyte proliferation

Studies have demonstrated that signaling pathways play a crucial role in inducing cardiomyocyte proliferation by influencing cell cycle regulators. These intricate molecular networks interact with each other to ensure precise control of the cell cycle in response to internal or environmental cues. Signaling pathways coordinate cellular responses and collaborate with transcription factors to regulate various cellular processes. Numerous pathways and proteins have been identified as key players in regulating cardiomyocyte proliferation following cardiac injury.

Hippo signaling pathway is evolutionarily conserved to control organ size and development by controlling proliferation, apoptosis, viability and differentiation [62, 130, 200]. The Hippo pathway in mammals comprises core components sterile 20-like protein kinases 1 and 2 (MST1/2), Salvador homolog 1 (Sav1), large tumor suppressors 1 and 2 (LATS1/2), MOB kinase activator 1A and 1B (MOB1A/1B), the two Yorkie homologs Yes-associated protein (YAP) and transcriptional coactivator with PDZ-binding motif (TAZ), and TEA domain family member (TEAD) [199]. Recent findings have revealed that Hippo pathway is essential for mammalian cardiomyocyte regeneration, along with its downstream effector Yes-associated protein (YAP)/ transcriptional co-activator with PDZ-binding motif (TAZ) [70, 112, 193]. Overexpression of YAP extends neonatal cardiomyocyte cell cycle activity and promotes proliferation [179], whereas cardiomyocyte-specific deletion of Yap impairs regenerative responses and the hearts display extensive cardiac fibrosis [193]. Based on the importance of YAP in regeneration, pharmaceutical interventions targeting YAP activity have significant potential for the treatment of heart failure. Nathaniel Kastan et al. have screened a small molecule inhibitor, TRULI, which can reversibly activate YAP and promote proliferation in postnatal day 0 (P0) murine cardiomyocyte in vitro [84]. Consistent with these results, cardiomyocyte-specific knockout of Sav, MST1/2 or LATS2 in mouse hearts also increases cardiomyocyte proliferation in the neonatal heart after adult myocardial infarction as well as after P8 myocardial infarction [70, 71]. Furthermore, knockdown of the Sav in border zone cardiomyocytes in pigs 2 weeks after I/R can significantly promote cardiomyocyte proliferation, reduce scar size and improve cardiac function without any tumor formation [114]. Taken together, Hippo pathway has great potential to be the clinical target for heart failure.

Phosphoinositide 3-kinase (PI3K)/protein kinase B (AKT) signaling pathway serves as one of the key mechanisms in cell cycle progression through regulating p21 and p27, cyclin D1 and CDK2 [118, 122, 132]. Studies in cardiomyocytes have also shown that PI3K/AKT pathway plays an important role in cardiomyocyte proliferation [121, 159]. Specific nuclear overexpression of AKT in mouse heart results in an increase in smaller cardiomyocytes, prolonged cell cycling and enhanced cardiac contractility [60, 151]. Mechanistically, PI3K/AKT pathway induces cardiomyocyte proliferation by coordinating with other signaling pathways to regulate downstream molecules. For example, acting as a downstream effector, PI3K subunit beta (PIK3CB) has been identified to mediate the effect of YAP on the activation of the PI3K-AKT pathway and thus the induction of CM proliferation [113]. As an upstream regulator, AKT phosphorylates and inhibits glycogen synthase kinase-3β (GSK-3β), which also plays central role in regulating Wnt signaling pathway. Deletion of GSK-3β in mice causes upregulated expression of GATA4, cyclin D1 and c-Myc, leading to cardiomyocyte hyperproliferation [85]. Cardiomyocyte-specific knockout of GSK-3β significantly promotes cardiomyocyte proliferation and preserves the cardiac function in mice after MI [202].

In addition, Jiang et al., have also shown that the Wnt/β-catenin signaling pathway can stimulate cardiomyocyte proliferation and cardiac development [81, 175]. In their study, activation of β-catenin decreases the ploidy and nuclear number of cardiomyocytes both in vitro and in vivo in mice, suggesting that β-catenin could promote cell division completion of polyploid and multinucleated cardiomyocytes. Similarly, overexpression of serine/threonine‐protein kinase 3 (SGK3) or its direct activator CDK9 can promote cardiomyocyte proliferation mainly through inactivating GSK-3β and upregulating β-catenin expression. As new potential therapeutic targets, overexpression of SGK3 or CDK9 after myocardial injury in mouse in vivo promotes cardiac repair and aims to partially restore the cardiac function [107, 166]. In addition, low-density lipoprotein receptor-related protein 5 (LRP5) and low-density lipoprotein receptor-related protein 6 (LRP6), co-receptors of Wnt signaling, are key regulators of cardiomyocyte cell cycle activity. Cardiac-specific knockout of LRP5 inhibits myocardial regeneration in the mouse after injury, whereas specific deletion of LRP6 in cardiomyocytes induces robust regeneration in mouse heart after MI and promoted recovery of cardiac function [191, 206].

The neuregulin-1 (NRG1)/ErbB signaling pathway, consisting mainly of growth factor NRG1 and its tyrosine kinase receptors ErbB2 and ErbB4, has been demonstrated to regulate cardiomyocyte proliferation [205]. Injection of recombinant NRG1, cardiac-specific overexpression of ErbB4 or transient overexpression of activated ErbB2 can all promote adult cardiomyocyte proliferation in mouse in vivo. Further studies in mouse disease models show that exogenous NRG1 or activated ErbB2 can induce cardiac regeneration, reduce the scar size and improve cardiac function by activating downstream PI3K or YAP after cardiac injury [3, 19].

The Notch signaling pathway regulates the activity of bone morphogenetic protein 10 (BMP10) and expression of NRG1, therefore it is essential for cardiomyocyte proliferation [55, 59]. Inhibition of Notch pathway impairs cardiac regeneration in zebrafish [204]; loss of BMP10 in mouse also leads to elevated expression of p57 and inhibited cardiomyocyte proliferation [33]. Consistently, activation of Notch pathway induces expression of cyclin D1 and cell cycle re-entry in quiescent neonatal murine cardiomyocytes [26]. However, hyperactivation of Notch signaling also blocks cardiomyocyte proliferation in zebrafish [204]. These findings indicate that the activity of targeted signaling pathway should be precisely controlled if it is to be used as therapeutic targets for cardiac regeneration. Summary of the information is shown in Table 2.

**Table 2:** Signal transduction pathways in cardiomyocyte regeneration and heart failure

Pathway (effector)	Species	Application	Effects	References
HIPPO (YAP)	Mouse Pig	OE or activation	CM proliferation ↑ Post-injury cardiac function ↑ Post-injury scar size ↓	[70, 71, 84, 114, 179, 193]
PI3K (AKT)	Mouse	OE	CM proliferation ↑	[60, 151]
Wnt (β-catenin)	Mouse	Activation	CM cytokinesis completion ↑ Post-injury cardiac function ↑	[81, 107, 166, 175]
NRG1/ErbB	Mouse	OE	CM proliferation ↑ Post-injury cardiac function ↑ Post-injury scar size ↓	[3, 19]
Notch	Mouse	Activation	CM cell cycle reentry ↑	[26]

*OE* overexpression; *CM* cardiomyocyte; *YAP* yes-associated protein; *PI3K* phosphoinositide 3-kinase; *AKT* protein kinase B; *NRG1* neuregulin-1

Apart from these canonical signaling pathways, many genes have been shown to promote cardiomyocyte re-entry into cell cycle, leading to cardiomyocyte proliferation. For example, overexpression of E2F1 or E2F2 in mouse heart induces cardiomyocyte cell cycle re-entry through regulating the expression of CDK4 or cyclin A and E respectively. However, overexpression of E2F1 leads to apoptosis [2], whereas E2F2 induces hypertrophic cell growth in cardiomyocytes [43]. Another important family of transcription factors in cardiomyocyte proliferation is T-box (TBX) gene family. Overexpression of TBX20 or TBX6 in adult mouse heart induces cardiomyocyte proliferation by regulating the expression of multiple cell cycle regulators, TBX20 overexpression also promotes cardiac repair and improves heart function in mouse after MI [61, 192]. A recent study reports that double knockdown of RB1 and Meis homeobox 2 (MEIS2) increased proliferation rate of human induced pluripotent stem cell-derived cardiomyocytes (hiPSC-CMs) in vitro and in rat in vivo [5]. Similarly, double knockout of Meis homeobox 1 (MEIS1) and HOXB13 in mouse in vivo can promote cardiomyocyte proliferation and improve cardiac function after MI [127]. As a deacetylase of p21, sirtuin1 (SIRT1) has been shown to positively regulate cardiomyocyte proliferation and protect cardiac function in mouse in vivo after MI via regulating p21 [99]. In addition, the anti-inflammatory cytokine interleukin 13 (IL-13) and the critical subunit of its type II receptor interleukin4Rα (IL4Rα) are also involved in regulating cardiomyocyte cell cycle and heart regeneration in neonatal mice [129, 187]. Another cytokine oncostatin M (OSM) and its receptor heterodimers oncostatin M receptor (OSMR)/glycoprotein 130 (gp130) are essential for cardiomyocyte proliferation, when activated can improve heart regeneration and function in mouse in vivo after MI [106]. Moreover, nuclear lamina filament Lamin B2 can promote cardiomyocyte M-phase progression and cytokinesis in neonatal mice [63]. Furthermore, knockout of the RNA binding protein muscleblind-like 1 (MBNL1) can promote cardiomyocyte proliferation in mouse heart after MI [14], and osteopontin (OPN) was found to enable cardiomyocyte re-entry into cell cycle in mouse heart, but also to stimulate other cells to improve scar formation and left ventricular remodeling [152].

### Manipulation of non-coding RNAs to induce cardiomyocyte proliferation

The ncRNAs are the functional RNAs without protein coding ability, including microRNA (miRNA), long non-coding RNA (lncRNA) and circular RNA (circRNA). These ncRNAs act as regulators to coordinate gene expression at the epigenetic, post-transcriptional, and translational levels [31]. A large number of ncRNAs have been reported to be associated with cardiomyocyte cell cycle regulation and cardiomyocyte proliferation [201].

miRNAs are small RNAs that bind to 3'-untranslated regions (3'-UTR) of mRNAs, leading to the degradation or repression of targeted mRNAs. Recent studies have shown that a range of miRNAs can induce cardiomyocyte proliferation by targeting the cell cycle regulators. A high-throughput functional phenotypic screen by using a whole-genome miRNA library has identified miR-199a, miR-302b, miR-518, miR-590 and miR-1825 among 204 potential human miRNAs that can increase 5-ethynyl-2'-deoxyuridine (EdU)-incorporation, Ki67, and pHH3 in mouse or rat cardiomyocytes [45]. Moreover, miR-199a and miR-590 have been linked to the activation of cell cycle induction and progression, resulting in the enhancement of cardiomyocyte proliferation in vitro and in vivo [45]. Further studies show that miR-199a directly targets two mRNAs, the upstream YAP inhibitory kinase TAOK1 and the E3 ubiquitin ligase β-TrCP, leading to YAP degradation [174]. Also, expression human microRNA-199a AAV serotype 6 (AAV6) in infarcted pig hearts can rescue and repair cardiac function via stimulating endogenous cardiomyocyte proliferation [53]. Another independent proliferation screen has been performed in hiPSC-CMs, and 96 miRNAs have been identified as drivers of DNA synthesis and cell division in cardiomyocytes, and 67 of the 96 miRNAs stimulate cardiomyocyte proliferation in a YAP-dependent manner [41]. Overexpression of miR-25 can increase the percentage of EdU and Ki67 positive cardiomyocytes by targeting FBXW7 and regulating cell cycle genes in human embryonic stem cell derived cardiomyocytes, as well as in zebrafish [180]. miR-106b ~ 25 cluster stimulates cardiomyocyte regeneration by targeting negative cell cycle regulators including E2F5, CDKN1C, CCNE1 and WEE1. Exogenous viral delivery of miR-106b ~ 25 induces cardiac regeneration and restores heart function after MI [146]. In addition, overexpression of miR-204 promotes cardiomyocyte proliferation in rat in vitro and mouse in vivo by targeting Jarid2 thus leading to the upregulation of cell cycle regulators Cyclin A, Cyclin B, Cyclin D2, Cyclin E, CDC2 and PCNA [109]. miR-499, a myocyte-specific miRNA (myomiR) expressed within one of the introns of β-myosin heavy chain (Myh7b) gene, can promote neonatal cardiomyocyte proliferation through regulating cyclin D1 and SRY-Box Transcription Factor 6 (Sox6) [104]. Similarly, miR-1825 is able to induce both DNA synthesis and cytokinesis in adult rat cardiomyocytes and improve cardiac function in vivo following MI, possibly by regulating the expression levels of miR-199a and its downstream targets RB1 and MEIS2 [131]. The studies provide the potential targets for gene therapy for cardiac regeneration in human patients.

In contrast, a large number of miRNAs have recently been identified that suppress the cell cycle in cardiomyocytes, such as miRNA let-7i-5p, miR-1/133a, miR-15 family, miR-26a, miR-29a/b, miR-34a and miR-128. miRNA let-7i-5p inhibits cardiomyocyte proliferation via targeting E2F2 and CCND2, while inhibition of let-7i-5p promotes mouse cardiomyocytes proliferation and enhance cardiac function after MI [74]. Similarly, miR-1/133a can prevent re-entry of adult rat cardiomyocytes into the cell cycle by suppressing FGFR1 and OSMR, while its inhibition promotes cardiomyocytes proliferation in mouse in vivo [176]. The miR-15 family (including miR-15a, miR-15b, miR-16-1, miR-16-2, miR-195 and miR-497), modulates cardiac regenerative capacity in neonatal mice and adult myocyte proliferation by regulating cell cycle genes and mitochondrial genes [139]. Overexpression of miR-195 impairs the cardiomyocyte proliferation and regenerative capability of P1 mouse heart after MI by repressing a number of cell cycle genes, including checkpoint kinase 1 (Chek1) in vivo [138, 139]. Inhibition of miR-26a enhances neonatal mouse cardiomyocyte proliferation through the regulation of cell cycle inhibitors in vitro and in vivo [39]. Overexpression of miR-29a suppresses cardiomyocyte proliferation, while its inhibition promotes cell division via upregulating the expression level of cyclin D2 in neonatal rat cardiomyocytes [28]. Similarly, inhibition of miR-29b promotes cardiomyocyte proliferation by targeting notch receptor 2 (NOTCH2) in mouse in vitro and zebrafish in vivo [195]. In addition, inhibition of miR-34a also results in enhanced cardiomyocyte proliferation and improved cardiac function after MI by regulating silent information regulator factor 2 related enzyme 1 (Sirt1), B-cell lymphoma 2 (Bcl2) and Cyclin D1 [196]. Likewise, inhibition of miR-128 promotes cardiomyocyte proliferation and improves cardiac function in response to MI through activating cyclin E and CDK2 [76].

lncRNAs are noncoding RNA molecules with nucleotides longer than 200, functioning through interactions with DNA, RNA or proteins. Similar to miRNAs, lncRNAs also engaged in the process of cardiomyocyte proliferation. As evidence, endogenous cardiac regeneration-associated regulator (ECRAR) was discovered to provoke myocardial regeneration and heart repair in rat in vivo after MI by activating cyclin D1 and cyclin E1 through E2F1-ECRAR-ERK1/2 signaling [35]. In addition, the downstream target of RNA-binding protein LIN28a, long noncoding RNA-H19, mediated the reprogramming of cardiomyocyte metabolism and enhancing of cell cycle activity, thereby protecting mouse heart from MI [150]. Conversely, cardiomyocyte proliferation regulator (CPR), LncDACH1 and natriuretic peptide A antisense RNA 1 (NPPA‑AS1) are recently reported to negatively regulate cardiomyocyte proliferation through binding and interacting with DNMT3A, PP1A/YAP1, splicing factor proline and glutamine rich (SFPQ), respectively. Deletion of CPR, LncDACH1 or NPPA-AS1 can restore cardiac function by promoting cardiomyocyte regeneration in mouse after MI [25, 52, 136]. These studies suggested lncRNA-based approaches as potential therapeutic treatments for heart failure.

circRNAs are stable RNA molecules formed closed loop structure by back-splicing. Although circRNAs are reported to be involved in regulation of many biological processes, the current scientific evidence supporting the role of circRNAs in regulating the cardiomyocyte cell cycle remains scarce. In the past years, only a few studies linked cicrRNAs to cardiomyocyte proliferation: overexpression of circNfix repressed cyclin A2 and cyclin B1 expression by inducing Y-box binding protein 1 (YBX1) ubiquitin-dependent degradation; it also increased miR-214 activity to promote GSK3β expression thus inhibiting cardiomyocyte proliferation. In contrast, downregulation of circNfix facilitated mouse heart regeneration and repair after MI [75]. Similar to circNfix, circMdc1 plays negative role in cardiomyocyte proliferation by blocking translation of MDC1 and when silenced can improve cardiomyocyte regeneration and heart function in vivo after injury [117]. The exploration of circRNAs in heart regeneration is still in the early stage, and deeper understanding of the molecular mechanisms underlying is required. Summary of the information is shown in Table 3.

**Table 3:** ncRNAs in cardiomyocyte regeneration and heart failure

ncRNAs	Species	Application	Effects	References
miR-199a, miR-302b, miR-518, miR-590	Mouse Rat	OE	CM cell cycle reentry ↑	[45]
miR-1825	Mouse Rat	OE	CM proliferation ↑ Post-MI cardiac function ↑	[45, 131]
miR-25	hiPSC-CM Zebrafish	OE	CM cell cycle reentry ↑	[180]
miR-106b ~ 25 cluster	Mouse	OE	CM cell cycle reentry ↑ Post-MI cardiac function ↑	[146]
miR-204	Mouse Rat	OE	CM cell cycle reentry ↑	[109]
miR-499	Rat	OE	Neonatal CM proliferation ↑	[104]
let-7i-5p	Mouse	Inhibition	CM proliferation ↑ Post-MI cardiac function ↑	[74]
miR-1/133a	Mouse	Inhibition	CM proliferation ↑	[176]
miR-195	Mouse	OE	CM proliferation ↓	[138, 139]
miR-26a	Mouse	Inhibition	Neonatal CM proliferation ↑	[39]
miR-29a	Rat	Inhibition	Neonatal CM proliferation ↑	[28]
miR-29b	Mouse Zebrafish	Inhibition	CM proliferation ↑	[195]
miR-34a	Mouse	Inhibition	CM proliferation ↑ Post-MI cardiac function ↑	[196]
miR-128	Mouse	Inhibition	CM proliferation ↑ Post-MI cardiac function ↑	[76]
ECRAR	Rat	OE	CM proliferation ↑ Post-MI cardiac function ↑	[35]
CPR	Mouse	KO	CM proliferation ↑ Post-injury cardiac function ↑	[136]
LncDACH1	Mouse hiPSC-CM	KO or KD	CM cell cycle reentry ↑ Post-MI cardiac function ↑ Post-MI infarct size ↓	[25]
NPPA‑AS1	Mouse	KO	CM proliferation ↑ Post-MI cardiac function ↑ Post-MI infarct size ↓	[52]
circNfix	Mouse	KD	CM proliferation ↑ Post-MI cardiac function ↑ Post-MI infarct size ↓	[75]
circMdc1	Mouse	KD	CM cell cycle reentry ↑ Post-injury cardiac function ↑	[117]

*OE* overexpression; *MI* myocardial infarction; *KD* knock down; *KO* knock out; *CM* cardiomyocyte; *ECRAR* endogenous cardiac regeneration-associated regulator; *CPR* cardiomyocyte proliferation regulator; *NPPA‑AS1* natriuretic peptide A antisense RNA 1; *circNfix* Circ_nuclear factor I X

Although initially considered as junk RNAs, more and more evidence highlights the indispensable role of ncRNAs in cellular processes including cardiomyocyte proliferation. Discovering potential ncRNAs targets in cardiac regeneration and understanding the mechanisms will help developing innovative strategies for heart regeneration.

### Manipulation of metabolism to induce cardiomyocyte cell cycle

During maturation, mammalian cardiomyocytes gradually exit the cell cycle and lose their regenerative potential, accompanied by a metabolic shift from glycolysis to fatty acid oxidation to meet the energy requirements of adult cardiomyocytes [115]. This highlights the critical role of cardiac metabolism in cardiac regeneration and further studies have identified potential metabolic targets that can be regulated to promote cardiomyocyte proliferation.

The metabolic shift during maturation suggests a negative correlation between cardiac regeneration and fatty acid oxidation, and a positive correlation with glycolysis. Despite the energetic advantage for adult cardiomyocytes, the increased production of ROS resulting from fatty acid oxidation can induce DNA damage and activate DNA damage response pathway, thus contributing to cardiomyocyte cell cycle arrest [123]. As reported by Cardoso et al., conditional knockout of pyruvate dehydrogenase kinase 4 (PDK4) in cardiomyocytes, which results in increased glucose relative to fatty acid oxidation, is able to reduce DNA damage and promote cardiomyocyte proliferation as well as cardiac function in response to MI in mouse in vivo [29]. In addition, inhibition of succinate dehydrogenase (SDH) by malonate promoted mouse cardiac regeneration after MI through stimulating metabolic switch to glycolysis [12]; knockdown of acyl CoA synthase long chain family member 1 (ACSL1), the key enzyme regulating lipid metabolism, can effectively induce myocardial regeneration after MI [108]. Recent study shows that blocking fatty acid oxidation in mouse cardiomyocytes by genetically deletion of carnitine palmitoyltransferase 1b (Cpt1b) reshapes epigenetic landscape thereby promoting cardiomyocyte proliferation and enabling cardiac regeneration after injury [105]. These studies further demonstrate that inhibition of the fatty acid utilization or acceleration of glycolysis can promote cardiomyocyte proliferation and provide potential therapeutic targets for heart failure.

Further, it has been reported that overexpression of pyruvate kinase muscle isozyme M2 (PKM2), an enzyme involved in the final step of glycolysis, using a novel cardiomyocyte-targeting strategy with modified RNA in mouse in vivo after MI can successfully promote cardiomyocyte cell division thereby improving cardiac function and long term survival [119]. Controversially, another study has shown that PKM2 inactivation in mouse heart promotes cardiomyocyte proliferation in the infarct zone after MI, thus indicating the opposite antiproliferative function of PKM2 [69]. Although more experiments are needed to clarify the function of PKM2, both of the studies show that PKM2 interacts with β-catenin to regulate cardiomyocyte proliferation, highlighting the crosstalk between metabolism and signaling pathway.

### Epigenetic regulation of cardiomyocyte cell cycle

In addition to the metabolic shift, an increase in global methylation was observed during rat heart development, suggesting a potential role of epigenetic modification in cardiomyocyte differentiation and proliferation [90]. As DNA methylome and histone modifications can regulate the chromatin condensation, resulting in differential chromatin accessibility and ultimately affecting the gene expression, epigenetics play an essential role in molecular networks and biological processes. In recent decades, increasing evidence has linked epigenetics to cardiomyocyte cell cycle re-entry.

N6-methyladenosine (m6A) is one of the most common modifications of messenger RNAs (mRNAs), and recent studies have shown that targeting m6A methylation can efficiently regulate cardiomyocyte re-entry into the cell cycle [64, 102, 207]. The knockdown of methyltransferase-like 3 (METTL3) has also been shown to reduce m6A-mediated pri-miR-143 maturation into miR-143-3p, thus promotes cardiac regeneration after MI through miR-143-YAP/CTNND1 (catenin delta-1) axis [57]; whereas forced expression of m6A demethylase ALKBH5 also promotes cardiac regeneration after MI in mice and CM proliferation in hiPSC-CM by improving the mRNA stability of YTH N6-methyladenosine RNA-binding protein 1 (YTHDF1) and consequently promoting the translation of YAP [64]. In addition, deletion of abraxas brother 1 (ABRO1) improved cardiac regeneration and mouse heart function after MI by targeting METTL3-mediated m6A methylation of PSPH mRNA and downstream CDK2 [182].

In contrast, mitochondrial transmembrane protein 11 (TMEM11)-mediated N7-methylguanosine (m7G) methylation negatively regulates cardiomyocyte proliferation and cardiac function in mouse in vivo after MI via targeting TMEM11-methyltransferase 1, tRNA methylguanosine (METTL1)-activating transcription factor 5 (ATF5)-inhibitor of CDK, cyclin A1 interacting protein 1 (INCA1) axis[34]. Several potential epigenetic targets for cardiomyocyte cell cycle regulation have also been recently reported. As shown by Paola Cattaneo et al., DOT1L was involved in cardiomyocyte cell cycle exit by catalyzing H3K79me2 and subsequently regulating transcriptional networks [30]. Moreover, knockdown of dual-specificity tyrosine regulated kinase 1A (DYRK1A) can enhance cardiomyocyte cell cycle activity via epigenetic modification of H3K4me3 and H3K27ac [95]. Furthermore, ablation of chromobox 7 (CBX7), a polycomb group (PcG) protein, can promote cardiac regeneration in both neonatal and adult injured hearts by targeting TAR DNA-binding protein 43 (TARDBP) and RNA binding motif protein 38 (RBM38) [38].

Taken together, the evidence to date highlights the importance of chromatin structure in cardiac cell cycle and identifies key factors with potential therapeutic value.

### Extrinsic factors driving the cardiomyocyte cell cycle

In the cellular context, cardiomyocytes reside in a complex and dynamic microenvironment with multiple cell types. Although the cardiomyocytes are the primary population and are responsible for the contractile function in the heart, proper cardiac function requires the cooperation of various cell populations. Recently, a number of extrinsic factors affecting cardiomyocyte cell-cycle arrest has recently been identified, including the oxygen concentration, extracellular matrix and the immune system.

The postnatal high oxygen level has been reported to be an important driver of mammalian cardiomyocyte cell cycle arrest after birth, mainly through the increase of reactive oxygen species (ROS) generated by the increased mitochondrial oxidative metabolism accompanied by a temporal shift from glycolytic to oxidative metabolism and the subsequent DNA damage response (DDR) [144]. On top of which, hypoxia or ROS scavenging can delay the cell cycle exit, thus prolonging the postnatal proliferative window of cardiomyocytes [144]. In adult mouse, gradual exposure to severe systemic hypoxemia (O_2_ is gradually decreased by 1% and maintained at 7% for 2 weeks) induces cardiomyocyte hypertrophy and promotes right ventricular cardiomyocyte proliferation [82], while in MI mouse, it promotes cardiac regeneration and thus improves cardiac function [126]. However, in the fetal rat heart, hypoxia (1% O_2_) inhibits cardiomyocyte proliferation [172]. In addition to this, maternal hypoxia (10.5% O_2_) decreases cardiomyocyte proliferation in both fetal and neonatal rat hearts [173]. The role of hypoxia in cardiomyocyte cell cycle regulation remains controversial. As shown by Ye et al., only the moderate range of hypoxia can promote cardiomyocyte cell cycle activities in both hiPSC-CMs and human heart samples [197]. They also showed that hypoxic treatment (10% O_2_) promotes the proliferation of neonatal rat cardiomyocytes, but inhibits cell cycle activities of fetal cardiomyocytes, suggesting that the role of hypoxia is related to development stage [168]. Taken together, the level of hypoxia and the phase or duration of cardiomyocytes experiencing hypoxia are key factors in regulating cardiomyocyte cell cycle.

The extracellular matrix (ECM), including structural proteins, matricellular proteins and carbohydrates, provides structural support to cells in the heart and mediates the cellular interactions, thus playing a vital role in regulating cell events [18, 49, 50]. A growing body of evidence suggests that the composition and rigidity of ECM are critical for the regulation of cardiomyocyte proliferation and cardiac regeneration [128, 164, 181, 183, 190]. It has been reported that the ECM proteins secreted by mouse embryonic cardiac fibroblasts including fibronectin, collagen and heparin-binding EGF-like growth factor, promote cardiomyocyte proliferation through β1 integrin and cell cycle-related genes [77]. Another separate study comparing the rat embryonic and postnatal cardiac fibroblast-derived ECM found that two embryonic ECM proteins SLIT2 and nephronectin (NPNT) can promote cardiomyocyte cytokinesis both in vitro and in vivo [190]. Individual ECM protein is also able to induce cardiac regeneration. For example, periostin has been shown to facilitate cardiomyocyte cell cycle re-entry and mitosis, thereby improving cardiac function after MI, in rat via activation of integrins and PI3K pathway [92]. However, the function of periostin in mouse remains controversial. In neonatal mice, knockout of periostin inhibits post-MI cardiac regeneration [36]; whereas in adult mice both overexpression and knockout periostin show no change in cardiomyocyte proliferation in the peri-infarct area [116]. Agrin, found in neonatal ECM, is able to drive hiPSC-CM proliferation and promote post-MI cardiac regeneration, thereby improving cardiac function via Yap signaling in both adult mice and pigs [13, 15]. Recently, the versican, a cardiac fibroblast–derived ECM protein, has been reported to promote hiPSC-CM proliferation. Intramyocardial injection of versican after MI results in reduced fibrosis and increased cardiomyocyte proliferation along with improved cardiac function in adult mice through activation of β1 integrin and downstream ERK1/2 and Akt [47]. These studies highlight the clinical potential of targeting ECM components for the treatment of heart failure. Meanwhile, ECM stiffness is also involved in the regulation of cardiomyocyte proliferation. As reported, compliant elastic matrices promote mouse cardiomyocyte dedifferentiation and cytokinesis in mice via regulating organization of the myoskeleton [194]. Correspondingly, decreased stiffness of ECM achieved by pharmacological inhibition of cross-linker enzyme lysyl oxidase (LOX) preserves the cardiac regenerative capacity in 3-day-old mice and enhances post-injury cardiac regeneration which induced by exogenous fetal ECM through increasing YAP localization to nuclei [128, 183].

In addition to fibroblast and ECM, the cardiac microenvironment consists of various cell types such as immune cells, adipocytes, vascular cells and neurons. Immune cells play an essential role in remodeling and inflammation after cardiac injury, and recent studies suggest that they also play an important role in cardiomyocyte proliferation. Specific ablation of CD4^+^ T-cells after MI by monoclonal antibody promotes cardiomyocyte proliferation in juvenile mice but not in adult mice [101]. Consistent with this, infarcted 1-day-old mice reconstituted with adult T cells have more monocyte-derived macrophage recruitment and show irreversible impairment of cardiac function along with increased fibrosis[42]. Furthermore, deletion of macrophages in neonatal mice blocks cardiac regeneration after MI [11]. Although immune cells show significant impact in regulating cardiomyocyte proliferation, the mechanisms involved remain unclear. Therefore, further efforts are needed to link immune cells to cardiomyocyte cell cycle. In addition, human adipose-derived stromal cells primed under hypoxic and pro-inflammatory conditions can facilitate the proliferation of rat neonatal cardiomyocytes via secreted interleukin-6 (IL-6) and activation of downstream Janus kinase-signal transducer and activator of transcription (JAK/STAT) and mitogen-activated protein kinases (MAPK) mitogenic pathways [142].

To date, studies of extrinsic factors show great clinical promise for the treatment of heart failure and highlight the importance of cardiac microenvironment which must be carefully considered when trying to develop interventional therapeutic strategies of cardiac regeneration.

## Different approaches of monitoring cardiomyocyte proliferation rate

Adult mammalian cardiomyocytes are terminally differentiated cells with limited mitotic activity. Similar to many terminally differentiated cell types, most of adult mammalian cardiomyocytes are polyploid or multinucleation [88, 97, 120]. Multiple genome copies resulted by polyploidization may enable cardiomyocytes to acquire elevated energy level thus increasing contractile force and enhance the tolerance to stress [9, 24, 97]. The polyploidization is induced by two different issues, karyokinesis failure and cytokinesis failure, during cell cycle [88]. Karyokinesis failure during which cell exit cell cycle without nuclear division results in polyploidy in single nuclear. While cytokinesis failure after successful karyokinesis generates multinucleated cell [24, 98].

Although a small proportion of the cardiomyocytes are able to enter cell cycle, the majority fail to complete karyokinesis or cytokinesis leading to polyploidization instead of authentic division into two new daughter cells [134]. Thereby, cardiac regeneration requires precise control of cardiomyocyte cell division and polyploidization. Even though numerous studies in the adult mammalian context have claimed successful regulation of cardiomyocyte re-entry into the cell cycle, the proliferation markers and indicators used have obvious limitations in identifying authentic cell division from polyploidization.

A commonly used approach is to measure the DNA synthesis activity by using thymidine analogs, such as bromodeoxyuridine (BrdU) or EdU, which is incorporated into the replicating DNA during S phase. Other common markers such as the nuclear protein Ki67 which present from G1 to M phase, and pHH3, present from G2 to M phase and responsible for chromatin condensation, are all limited in that they do not indicate the completion of cytokinesis [8]. Thus, these assays could detect the DNA synthesis, but could not distinguish between the multinucleation, polyploidization, or de novo generation of cardiomyocytes. However, cardiomyocyte DNA replication often results in a polyploid cardiomyocyte and only rarely leads to a new cardiomyocyte by cell division. Besides, Aurora B is a quantifiable marker for cardiomyocytes that is ultimately localized to the cleavage furrow during cytokinesis [10]. However, it also has significant shortcomings. On the one hand, it is a very rare event with a short window of opportunity, only about 0–0.04% of adult mammalian cardiomyocytes are Aurora B positive as shown by in vivo studies [184, 192]. The extremely low proportion of Aurora B-positive cardiomyocytes makes it extremely difficult to quantify the proliferation rate. The complexity of cardiac tissue also makes it difficult to identify the cytokinetic furrow and associated cells in vivo. On the other hand, Aurora B-positive cardiomyocytes may also fail to complete cytokinesis, leading to multinucleation, as indicated by the position of midbody and the distance between the daughter nuclei [44, 73]. An additional marker, anillin, is necessary in this setting to assess if cardiomyocytes form multi-nucleated cells or complete the cell cycle through formation of daughter cells. Multinucleation is characterized by the asymmetric constriction of the cleavage furrow and defective midbody formation, as indicated by Aurora B and Anillin colocalization [44, 98]. In conclusion, each of the markers used so far has served as an indicator of cell cycle activity, but none of them alone can be considered as definitive indicators of authentic cell division but not polyploidization in cardiomyocytes.

To date, many transgenic reporter mice have been generated based on tracking these proliferative indicators for real-time cell cycle monitoring. Kretzschmar et al. generate a mouse model to label Ki67 in cycling cells with red fluorescent tdTomato [91]. In addition, Raulf et al. generate the double transgenic Myh6-H2B-mCherry/CAG-eGFP-Anillin transgenic mouse. By labeling cardiomyocyte nuclei with red fluorescence and Anillin with green fluorescence, the mouse model is able to show the kinetic localization of Anillin and thus indicate the proliferation status of cardiomyocytes [147]. Moreover, an Aurora B reporter–based mouse system with a tdTomato fluorescence labeling to monitor the proliferating cardiomyocytes has also been generated [51, 80]. However, these models using a single proliferative marker is not sufficient to quantify the authentic cytokinesis in cardiomyocytes.

In addition to these mouse systems, fluorescent ubiquitination-based cell cycle indicator (FUCCI) system has been specifically developed to study cell cycle dynamics and the cardiomyocyte proliferation. Cardiomyocyte-specific FUCCI system consists of two different fluorescent reporters driven by the α-myosin heavy chain (αMHC) promoter and relies on the ubiquitination and degradation of DNA replication factor Cdt1 and Geminin [8]. The FUCCI system allows the assessment of cycling and non-cycling cells, but the approach is limited by the rare activation of cycling cardiomyocytes in adult hearts after cardiac injury and the inability to label daughter cells to identify endoreplicative and authentic cell division in vivo*.* In addition, another mouse model, mosaic analysis with double markers (MADM) system, has been developed to label cardiomyocytes that have completed cytokinesis. MADM system requires introduction of two reciprocal chimeric marker genes, each consisting of part of green and red fluorescent protein coding sequence separated by loxP site, to identical loci on homologous chromosomes. Based on Cre-mediated recombination between loxP sites, 4 types of daughter cells are possible after mitosis including single-colored green, single-colored red, double colored yellow and colorless cells [208]. If mitotic recombination occurs during G0 or G1 phase, a double-labeled cell is produced without changing the genotype of the cells, only completed cytokinesis is capable of producing single-colored daughter cells, making MADM a gold standard for lineage tracing in cardiomyocyte proliferation. Despite potential limitations of this model, such as Cre combination efficiency and the individual variability, MADM remains a powerful tool for investigating cardiomyocyte proliferation.

In conclusion, these newly developed systems allow distinguishing between authentic cell division and polyploidization thus offering an opportunity to validate previous research on the proliferation of cardiomyocytes. However, it is important to acknowledge the limitations of using only proliferative markers and address potential challenges by utilizing a combination of different approaches.

## Challenges in cardiac regeneration research

The ultimate goal of improving cardiac regeneration is to repair damaged heart and improve cardiac function by increasing the number of cardiomyocytes. Although much effort has been put into understanding cell cycle regulation and gene or cell therapy to induce cardiomyocyte proliferation, therapeutic cardiac regeneration has not yet been achieved. Before translation, there are still several conceptual and technical challenges needed to be addressed to ensure the safety and efficacy of the gene therapy for the treatment of heart disease.

Most studies to date have only utilized in vitro or small rodent models to study cardiomyocyte cell cycle regulation. However, despite their homology, there are significant differences between human and rodent genomes. The mouse genome is approximately 14% smaller than the human genome with approximately 20% of genes having no identified orthologue in human genome, especially non-coding genes [125]. To bridge the gap between rodents and humans and to advance preclinical translational studies for cardiac regeneration, large animal models, such as dogs, pigs, sheep and non-human primates are an indispensable step in translating basic research into clinical applications. So far, only the CCNA2, CCND2, YAP, Sav and miR-199a have been reached in pig studies [13, 53, 114, 157, 165]. Therefore, further investigations are needed to validate the potential therapeutic effects of the targets in large animal models before clinical translation. However, also need to be noted that, large animals such as pigs also have obvious limitations as preclinical research models for human translational medicine. Despite the similarities in terms of organ size and physiology of pig and human, they are different in heart development, anatomy and genome, especially ncRNAs [135, 156, 178]. Thus, the impact of regenerative strategies being tested in the pigs needs to be considered, but the use of the pig system for studies of cardiac regeneration still offers inspiring and exciting possibilities to improve clinical translation to humans.

In addition, the molecular cell cycle control system of cardiomyocytes involves proto-oncogenes and tumor suppressors, and regulation of these genes to induce cell cycle re-entry may result in uncontrolled cardiomyocyte proliferation and even tumorigenesis, and also may lead to cardiac dysfunction in the long term [67, 177]. For example, transient overexpression of Oct4, Sox2, Klf4, and c-Myc (OSKM) in adult mouse cardiomyocytes induces cell cycle re-entry and promotes non-tumorigenic cardiac regeneration, whereas prolonged expression of OSKM leads to cardiac tumor formation [36]. Despite promising improvement of gene therapy in cardiac regeneration, these studies have been limited by tumor formation, strong immunological response, and high mortality rate. Thus, the precise control of cardiomyocyte proliferation with respect to reversibility is required to prevent tumor formation and maintain cardiac function, and is critical for the advancement of gene therapy in the clinical setting.

In addition, the adult cardiomyocytes tend to fail karyokinesis or cytokinesis resulting in polyploidy or multinucleation during the cell cycle. Studies have shown that the transcription levels are different between the diploid and polyploid cardiomyocytes [186, 198]. Furthermore, recent studies have shown that polyploid and multi-nucleated cardiomyocytes are also able to re-enter into cell cycle and complete cell division [53, 169]. Therefore, it is important to investigate the molecular mechanisms regulating cardiomyocyte cell division and polyploidization in cardiac regeneration prior to clinical translation studies. Currently the single-cell RNA sequencing (scRNA-seq) can potentially characterize the individual cells and elucidate the biological mechanism at the cellular level, but it still does not have the sufficient depth to reveal all differences at the single cell level. Thus, the further studies including electromechanical coupling and contractile function are needed, and new tools also need to be developed.

Moreover, the efficiency of RNA delivery directly related to the efficacy of gene therapy. Therefore, the specific while efficient gene delivery to the heart is fundamental for the clinical application of cardiomyocyte regeneration. Most of the studies discussed above have relied on adeno-associated virus (AAV), ultrasound-targeted microbubble destruction (UTMD) which combines ultrasound-mediated delivery with microbubbles, exosome-mediated delivery or nanoparticle delivery [46, 149, 155, 203]. However, RNA delivery has the limitation of low gene transduction, and have resulted in orthotropic delivery of therapeutics into the myocardium via catheter or intracardiac injection, limiting the potential accessibility of such therapeutics [79]. Therefore, optimization of the delivery method and reduction of the off-target effects for gene therapy need to be addressed in the further investigations.

Finally, most studies are performed in young and healthy animals. However, heart failure in humans mostly affects older adults. Aging is also associated with an increase in deaths from heart disease [160]. Therefore, it is really important to know if the regenerative therapy can induce cardiomyocyte proliferation in aged animals as it does in young adults. Besides, experiments in chronic heart failure models are also required. Although in vivo studies show cardiomyocyte regeneration, almost all in vivo studies have been performed in the acute stage of MI. It remains unknown whether the therapy could be applied to chronic heart failure models. Therefore, the current regenerative therapy in the aged animals and chronic heart failure models need to be addressed before clinical setting.

In summary, although many challenges remain, the clinical application of cardiomyocyte proliferation induction by regulating cell cycle related targets may offer a bright future for the treatment of heart diseases.

## Conclusion

The massive loss of cardiomyocytes after cardiac injury and their subsequent replacement by fibrotic tissue is the major cause of heart failure, while the cardiomyocytes have limited proliferative capacity. Therefore, inducing resident cardiomyocytes to re-enter the cell cycle and progress through mitosis and cytokinesis is the most physiological approach to achieve cardiomyocyte regeneration with improved cardiac function in the diseased heart. In this review, we have discussed several aspects targeting cardiomyocyte regeneration via cell cycle regulation. While research in these areas is promising, challenges remain in controlling authentic cardiomyocyte cell division and translating these approaches into clinical practice. In addition, a combinatorial approach to induce cardiomyocyte regeneration may need to be developed to overcome the current limitations and challenges for therapeutic cardiac regeneration in patients in the future.
